# Design and Implementation of a Subnanometer Heterodyne Interference Signal Processing Algorithm with a Dynamic Filter

**DOI:** 10.3390/s22145422

**Published:** 2022-07-20

**Authors:** Qilin Zeng, Zhengyi Zhao, Xianming Xiong, Hao Du, Wentao Zhang, Zhicheng Zhang, Peng Wang, Lihua Lei

**Affiliations:** 1 Department of Electrical Engineering and Automation, Guilin University of Electronic Technology, Guilin 541004, China; qilinzeng@guet.edu.cn (Q.Z.); 20082304075@mails.guet.edu.cn (Z.Z.); xmxiong@guet.edu.cn (X.X.); duh@guet.edu.cn (H.D.); 19082304040@mails.guet.edu.cn (Z.Z.); 19082202012@mails.guet.edu.cn (P.W.); 2 Key Laboratory of Optoelectronic Information Processing, Guilin University of Electronic Technology, Guilin 541004, China; 3 Shanghai Key Laboratory of Online Test and Control Technology, Shanghai 201203, China; leilh@simt.com.cn

**Keywords:** dynamic filter, precision measurement, electronic segmentation, heterodyne interference signal processing algorithms

## Abstract

In this study, a subnanometer heterodyne interference signal processing algorithm with a dynamic filter is proposed. The algorithm can effectively reduce the measurement error caused by the noise introduced in the optical path and circuit. Because of the low signal−to−noise ratio of the measurement signal, a dynamic filter with variable coefficients is designed. The role of the bi−quadrature lock−in amplifier algorithm in the problem of different amplitudes among the measurement signal, reference signal, and uncertainty of the frequency difference of the dual−frequency laser is analyzed. With the aid of the heterodyne interferometry platform, the error in the solution results of the proposed algorithm and the conventional algorithm is compared. The results indicate that the maximum deviation of the phase increment of the algorithm does not exceed 6 mrad, the single−cycle phase difference can be subdivided by 1024, and the system resolution reaches 0.15 nm.

## 1. Introduction

Advancement in the information technology industry has increased the demand for high−end semiconductors. To meet the process and performance requirements of chip manufacture, the measurement accuracy of the lithography machine’s displacement system must reach the subnanometer level [1]. At present, the measurement tools used in the field of ultraprecision displacement measurement mainly include heterodyne laser and grating interferometers [2,3,4,5,6].

By subdividing the phase difference between the measurement and reference signal, the resolution can be greatly improved without changing the optical path structure. Increasing the number of subdivisions by hardware frequency multiplier will be limited by the chip device [7]. At present, the phase subdivision method based on amplitude resolution is mostly used to further improve the resolution. Many types of phase subdivision methods are based on amplitude resolution, all of which should use an avalanche photon diode (APD) to convert light intensity signal into an electrical signal and then convert it into a digital signal through an analog−to−digital converter (ADC) [8].

During the measurement process, since the measurement signal passes through many optical devices, the strength and signal-to-noise ratio of the measurement signal are low relative to the reference signal. Due to the increased demand for measurement speed, the frequency variation of the measurement signal is larger. When a general band−pass filter is used for signal preprocessing, the pass-band is large in order to pass the measurement signal with a large frequency variation range. A large passband will make it difficult for ordinary filters to suppress unwanted frequency components well.

Meanwhile, as shown in Equations (1) and (2), the ideally measured and reference signal amplitudes are R and M, respectively. The frequency difference of the dual−frequency laser is not ideal $f_{1}-f_{2}$ but has an uncertainty $f^{\prime }$. This brings certain difficulties to the subsequent phase calculation, where Δf is the frequency change of the measured signal caused by Doppler frequency shift.

(1) $$\begin{array}{cc} f_{r}= & R\sin[2\pi \left(f_{1}-f_{2}+f^{\prime }\right)t] \end{array}$$

(2) $$\begin{array}{cc} f_{m}= & M\sin[2\pi \left(f_{1}-f_{2}+f^{\prime }+\Delta f\right)t] \end{array}$$

This study did the following:

1. Aiming at the problem that the signal−to−noise ratio of the measured signal is low and the phase error after the solution is large, a sub−nanometer heterodyne interference signal processing algorithm with a dynamic filter is proposed. The center frequency of the passband can change with the main frequency. On the one hand, it can ensure that the measurement signal of 4–36 MHz passes through the filter. On the other hand, it can keep the passband narrow and attenuate most of the unwanted frequency components, which improves the system’s resolution.
2. Aiming at the problems of different amplitudes of reference and measurement signals and the uncertainty of the frequency difference of the dual−frequency laser, the function of the phase solution algorithm of bi−quadrature lock−in amplification in solving these two kinds of problems is analyzed. The bi−quadrature lock−in amplification algorithm can solve these two types of problems in principle by mixing, filtering and phase difference, and finally generate two quadrature signals with equal amplitudes and only containing phase information [9].
3. Several groups of experiments are designed to compare the solution effect of the general bandpass filter and heterodyne interference signal processing algorithm with a dynamic filter. A hardware−in−the−loop simulation experiment is designed to test the solution effect of the phase solution algorithm with a dynamic filter when the motion speed and acceleration of the motion table are large. It is proved that in the frequency range of 4–36 MHz, the filter can attenuate the unwanted frequencies well, thereby improving the resolution of the system.

## 2. Signal Processing Algorithm Design

**Figure 1:**
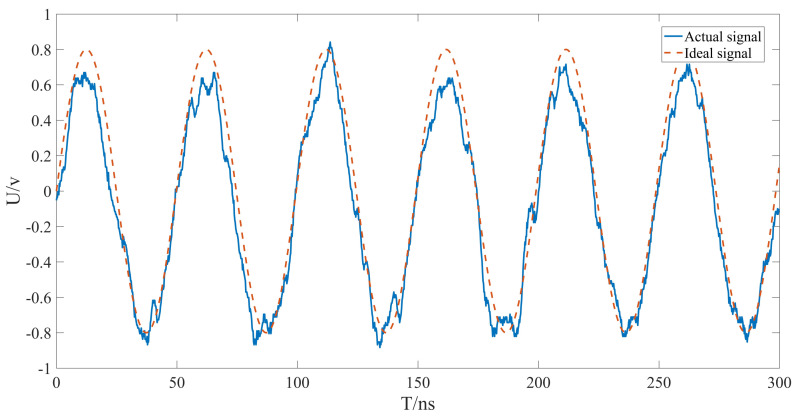
Comparison between the measured signal after photoelectric conversion and the ideal signal.

Although the light intensity signal output by the heterodyne interferometry platform is a sinusoidal signal, other noises will be introduced in the optical path, APD, signal conditioning, and analog−to−digital conversion [10]. This is especially evident in the measurement signal, as shown in Figure 1.

Additionally, the frequency difference of the dual−frequency laser is not an ideal signal of a specific frequency but has an uncertainty $f^{\prime }$. Furthermore, there is a difference between the amplitudes of the two signals. This is because the optical paths and circuits that the measurement and reference signal pass through are different [11].

**Figure 2:**
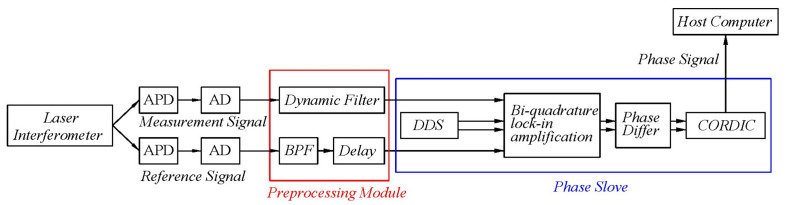
Block diagram of subnanometer heterodyne interference signal processing algorithm with a dynamic filter.

To solve these problems, a subnanometer heterodyne interference signal processing algorithm with a dynamic filter is proposed. To verify the proposed algorithm, a heterodyne interferometric measurement system, as shown in Figure 2, is built. After the dual−frequency laser passes through the beam splitter, it is divided into reference light and measurement light. The reference light passes through the polarizer and interferes to form a reference signal. The measurement light forms the measurement signal through the four−subdivided laser interferometric structure. The measurement and reference signals first go through the preprocessing module, which improves the signal quality through dynamic filtering. They further pass through the phase solution module, where the problems of the uncertainty of the dual−frequency laser and the different amplitudes of the two signals are solved. Finally, the Coordinate Rotation Digital Computer (CORDIC) algorithm solves the phase signal.

### 2.1. Preprocessing Module Design

**Figure 3:**
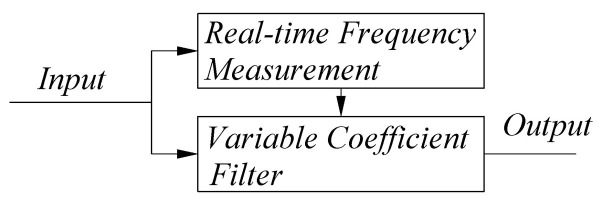
Block diagram of the preprocessing module.

Because the ideal measurement signal is a sinusoidal signal of a single frequency, components of other frequencies except for the main frequency of the measurement signal can be regarded as noise. In order to minimize the effect of noise, it is necessary to make the passband bandwidth of the filter narrow. However, because the frequency variation range of the measurement signal is 4–36 MHz, in order to make the measurement signal pass through the filter while maintaining a narrow passband, a variable coefficient dynamic filter as shown in Figure 3 is designed. The frequency real−time calculation module can calculate the frequency of the input signal in real−time, and the variable parameter filter module changes the filter parameters according to the frequency of the current signal so that the center frequency of the passband moves with the main frequency.

**Figure 4:**
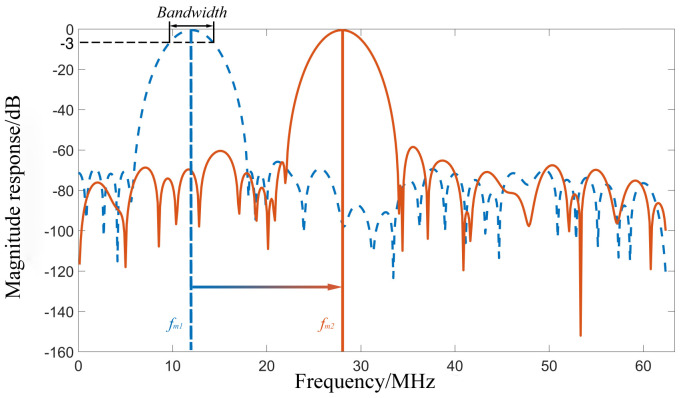
Diagram of the filter passband change.

As shown in Figure 4, when the Doppler frequency shift changes, the frequency of the measurement signal changes from $f_{m1}$ to $f_{m2}$ and the center frequency of the filter passband also changes accordingly, while ensuring that all useful signals can pass through the filter, the passband bandwidth becomes narrow. Hence, the noise blocking effect is better than the general bandpass filter.

#### 2.1.1. Design of Real−Time Frequency Measurement Module

To make the center frequency of the filter passband follow the frequency of the measurement signal, it is first necessary to measure the accurate measurement signal frequency with a high update rate. In the normal method for counting and measuring cycles, the update rate is associated with the frequency of the measurement signal, whereas the measurement accuracy depends on a high−frequency and high−precision clock.

**Figure 5:**
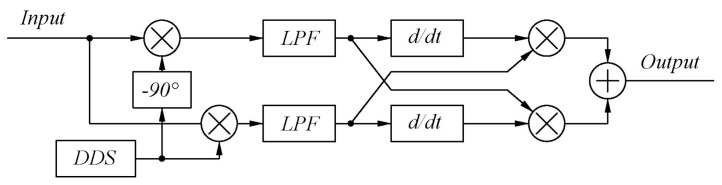
Diagram of the real−time frequency calculation module.

Given the aforementioned problems, the following real−time frequency calculation part, which is based on the automatic frequency control differential loop structure, is designed and its structure is shown in Figure 5 [12].

The input measurement signal is a sinusoidal signal with a frequency of $f_{1}-f_{2}+\Delta f$. The expression is shown in Equation (3), where M is the measured signal amplitude, $f_{1}-f_{2}$ is the dual−frequency laser frequency difference, and Δf is the frequency change of the measured signal caused by Doppler frequency shift.

(3) $$f_{m}=M\sin\left[2\pi \left(f_{1}-f_{2}+\Delta f\right)t\right]$$

A direct digital frequency synthesizer (DDS) can be implemented using a field−programmable gate array (FPGA) to generate a fixed frequency signal. The two−way signals it generates are shown in Equations (4) and (5), where $f_{d}$ is the frequency of the DDS generated signal.

(4) $$\begin{array}{c} f_{dds\;sin}=2\sin\left(2\pi f_{d}t\right) \end{array}$$

(5) $$\begin{array}{c} f_{dds\;cos}=2\cos\left(2\pi f_{d}t\right) \end{array}$$

These two signals are multiplied by the measurement signal $f_{m}$ to obtain two mixing signals as shown in Equations (6) and (7). The two mixed signals are the sum of a high−frequency signal with the frequency $f_{1}-f_{2}+\Delta f+f_{d}$ and a low−frequency signal with the frequency $f_{1}-f_{2}+\Delta f-f_{d}$.

(6) $$\begin{array}{c} f_{m\times sin}=M\sin\left[2\pi \left(f_{1}-f_{2}+\Delta f-f_{d}\right)t\right]+M\sin\left[2\pi \left(f_{1}-f_{2}+\Delta f+f_{d}\right)t\right] \end{array}$$

(7) $$\begin{array}{c} f_{m\times cos}=M\cos\left[2\pi \left(f_{1}-f_{2}+\Delta f-f_{d}\right)t\right]-M\cos\left[2\pi \left(f_{1}-f_{2}+\Delta f+f_{d}\right)t\right] \end{array}$$

After passing through the low−pass filter (LPF), whose cutoff frequency is between $f_{1}-f_{2}+\Delta f_{max}-f_{d}$ and $f_{1}-f_{2}+\Delta f_{min}+f_{d}$, the high−frequency part is filtered out, and the low−frequency part is shown in Equations (8) and (9). $\Delta f_{max}$ and $\Delta f_{min}$ are the maximum and minimum values of Δf, respectively.

(8) $$\begin{array}{c} f_{m\times sin\;low}=M\sin\left[2\pi \left(f_{1}-f_{2}+\Delta f-f_{d}\right)t\right] \end{array}$$

(9) $$\begin{array}{c} f_{m\times cos\;low}=M\cos\left[2\pi \left(f_{1}-f_{2}+\Delta f-f_{d}\right)t\right] \end{array}$$

After differentiation and cross−multiplication, the signals in the form of two sums of squares, as shown in Equations (10) and (11), will be obtained. Subtract the two formulas to get the final result, and the expression of the final result Fd is shown in Equation (12).

(10) $$f_{m\times sin\;low}\times f_{m\times cos\;low}^{\prime }=M^{2}\left[2\pi \left(f_{1}-f_{2}+\Delta f-f_{d}\right)\right]\sin^{2}\left[\left(f_{1}-f_{2}+\Delta f-f_{d}\right)t\right]$$

(11) $$f_{m\times cos\;low}\times f_{m\times sin\;low}^{\prime }=-M^{2}\left[2\pi \left(f_{1}-f_{2}+\Delta f-f_{d}\right)\right]\cos^{2}\left[\left(f_{1}-f_{2}+\Delta f-f_{d}\right)t\right]$$

(12) $$Fd=2\pi M^{2}\left(f_{1}-f_{2}+\Delta f-f_{d}\right)$$

Because the maximum speed of the motion stage is the same in forward and reverse motion, the maximum and minimum values of the Doppler frequency shift Δf are equal in value and opposite in sign. Set the frequency $f_{d}$ of the dds signal to be equal to the frequency difference $f_{1}-f_{2}$ of the dual−frequency laser. Since the order of magnitude of the frequency error of the signal generated by dds and the frequency stability of the dual−frequency laser are both in the order of Hz, the influence on the subsequent calculation results can be ignored, so $f_{d}$ and $f_{1}-f_{2}$ can be considered to be equal. The variation range of the output result can be made symmetrical at approximately 0, with the bit width being the smallest when calculating in the FPGA, saving the internal resources of the FPGA. Its expression is shown in Equation (13).

(13) $$Fd=2\pi M^{2}\Delta f$$

**Figure 6:**
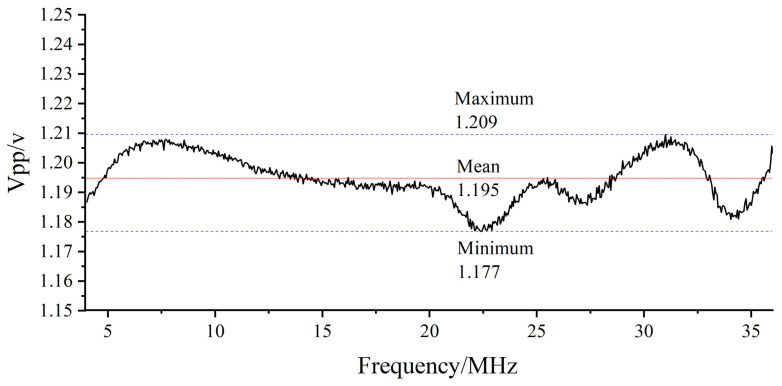
The relationship between the frequency and peak−to−peak value of the measured signal voltage after photoelectric conversion.

Because of the hardware’s automatic gain compensation design [13,14], the peak−to−peak variation in the entire signal frequency band is within ±18 mV, which can be ignored. The corresponding relationship between the frequency and peak−to−peak value of the signal is shown in Figure 6.

The frequency difference of the dual−frequency laser used in this study is 20 MHz. The design requires a maximum measurement speed of 2.5 m/s, and the corresponding maximum and minimum Doppler frequency shifts are $\Delta f_{min}$ = −16 MHz and $\Delta f_{max}$ = 16 MHz, respectively. The cutoff frequency of the LPF can be selected in the range of 16–24 MHz, with 16 MHz selected as the cutoff frequency in this study.

**Figure 7:**
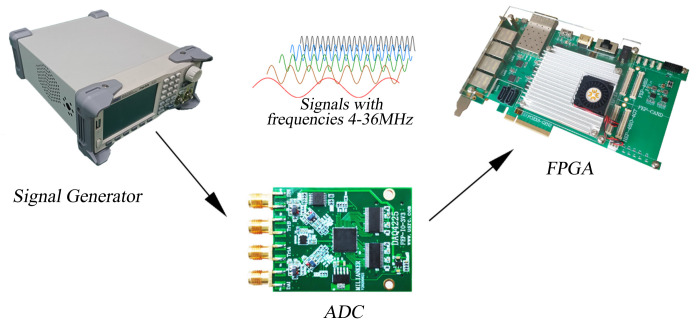
Diagram of a hardware−in−the−loop simulation system.

The measurement system itself has errors; it is difficult for the motion stage to maintain a high-precision uniform motion for a long time; hence, the input signal cannot be stabilized at a certain frequency. Therefore, to test the performance of the real−time frequency measurement part, a hardware−in−the−loop simulation platform as shown in Figure 7 is built.

**Figure 8:**
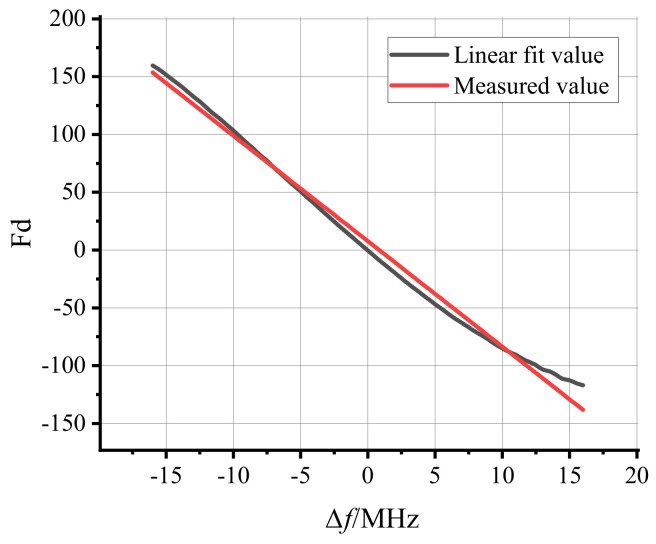
The relationship between the output result of the frequency real-time calculation module and the measured signal frequency.

The measurement signal is a 4–36 MHz sine wave, and a signal generator is used to simulate the measurement signal. The signal is collected using an ADC and sent to the FPGA for calculation. Through the appropriate truncation of the result, the change in the result is within 1 LSB. The frequency test is performed at every 0.5 MHz in the range of 4–36 MHz, and the average value is calculated by taking 8192 consecutive numbers. The result is shown in Figure 8.

After calculation, the mean square error and the root mean square values were 57.77 and 7.72, respectively, which are small. The value of the coefficient of determination (R2) is 0.9922, which is very close to 1. The real−time frequency measurement system has better performance and less error than the traditional method for measuring period and frequency. The update speed is not affected by the frequency of the input signal, and each sampling period can be updated through the pipeline design. Furthermore, the accuracy improvement does not depend on the high−frequency clock.

#### 2.1.2. Variable Coefficient Filter Design

According to the measured frequency, the coefficient of the filter can be dynamically changed, so that the center frequency of the filter’s passband moves with the measured signal. However, in the process of filter coefficient change, it is difficult to ensure that neither the phase nor the amplitude of the signal undergoes abrupt changes.

**Figure 9:**
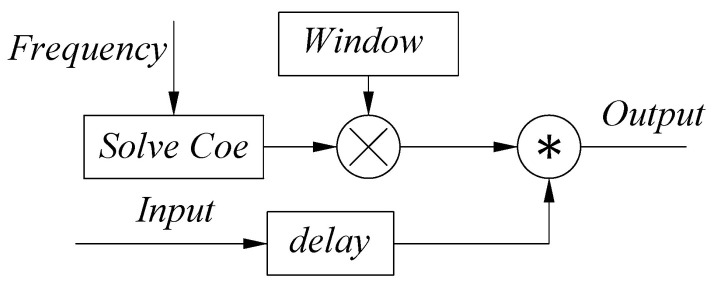
Diagram of a variable coefficient filter block.

By focusing on the aforementioned problems, a variable coefficient filter based on a window function as shown in Figure 9 is designed.

Digital filters can be designed in many ways [15,16,17,18]; the simplest and most widely used method used method for designing a linear phase response digital filter is via the Fourier series method using a window function [19]. When the frequency of the measured signal is known, the frequency of the measured signal is taken as the center frequency of the filter passband. Once the bandwidth is determined, the high and low cutoff frequencies of the filter can be calculated. Then, according to Equation (14), the filter coefficients before windowing can be calculated. In the formula, M is the filter order and $f_{l}$ and $f_{h}$ are the low and high cutoff frequencies of the band−pass filter, respectively, [20].

(14) $$h_{d}\left(n\right)=\frac{\sin\left[2\pi f_{h}\left(n-\frac{M}{2}\right)\right]-\sin\left[2\pi f_{l}\left(n-\frac{M}{2}\right)\right]}{\pi (n-\frac{M}{2})},n=1,2\cdots ,M$$

To reduce the ripple and narrow the transition band, the filter coefficients need to be windowed after comprehensive consideration of stopband attenuation, main lobe, and transition bandwidth. The Kaiser window is used as the window function, and the expression of the Kaiser window is shown in Equation (15). $I_{0}$ is a modified Besso function of the first kind of zeroth order. β is an arbitrary non−negative real number used to adjust the shape of the Kaiser window.

(15) $$w_{k}\left(n_{k}\right)=\frac{I_{0}\left[\beta \sqrt{1-{\left(1-\frac{2n_{k}}{N-1}\right)}^{2}}\right]}{i_{0}\beta },0\leq n_{k}\leq N-1$$

The value of β can be adjusted according to the empirical Equation (16), where $A_{s}$ is the stopband attenuation. When β = 8 and the order is 64, the stopband attenuation can reach 60 dB.

(16) $$\beta =0.1102(A_{s}-8.7)$$

**Figure 10:**
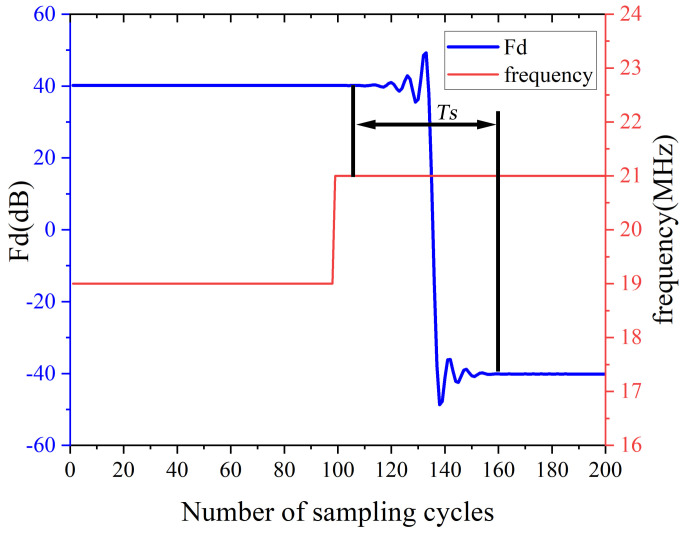
Changes in the output of the frequency real−time calculation module when the frequency suddenly changes.

Theoretically, the narrower the bandwidth of the variable coefficient dynamic filter, the higher the signal−to−noise ratio of the obtained signal. However, when the frequency changes, it takes some time for the measured results of the real−time frequency measurement system to stabilize. During this period, if the frequency changes significantly and exceeds the bandwidth, the measurement signal will be suppressed. To test the response time, the input signal is abruptly changed from 21 to 19 MHz and the response speed of the aforementioned frequency real−time calculation module is tested. The results are shown in Figure 10. $T_{s}$ represents the time taken for the output of the frequency measurement system to change from beginning to stable, and the value is equal to 312.5 ns. The maximum acceleration of the motion table in the experimental setup is 32 m/s${}^{2}$. The laser interferometer is used for the measurement of displacement, and when the measured object runs at the maximum acceleration within $T_{s}$, the corresponding frequency change is 16 KHz. If the passband bandwidth is greater than 32 KHz, the useful signal can always pass through the filter during the equilibrium time. If the bandwidth is too narrow, although the signal quality will be better, it will take up a significant amount of the FPGA resources. Furthermore, before the measurement signal is processed, the noise contained will cause a certain error in the measured frequency. After weighing the internal resource occupation of the FPGA and the signal−to−noise ratio, select a 4 MHz bandwidth and set it to gear at every 1 MHz. The center frequency of the passband is set to the integer frequency closest to the frequency of the measured signal, whereas the coefficients of the gears are calculated in advance and stored in the FPGA.

**Figure 11:**
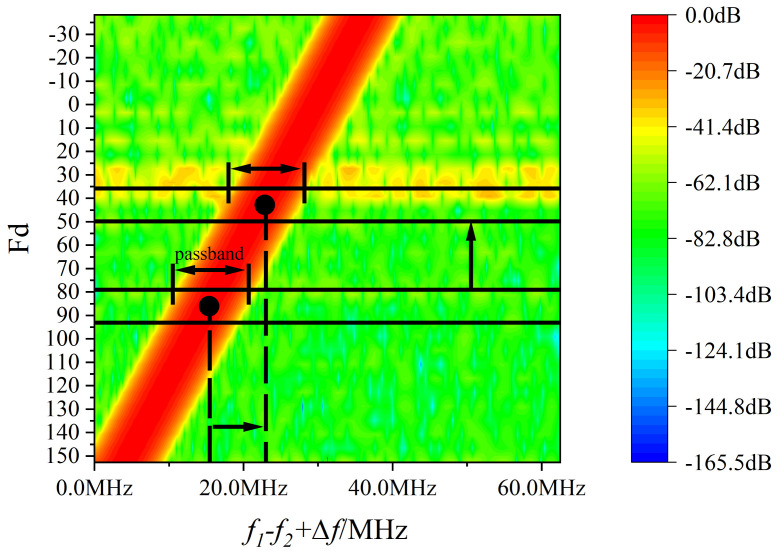
Frequency response of the variable coefficient filter.

The designed filter frequency response is shown in Figure 11. The filter coefficients are determined from the value of Fd, and the frequency response is determined accordingly. The filter passband is just enough to pass the signal at the current frequency. As the frequency of the measurement signal changes, the frequency response also changes.

**Figure 12:**
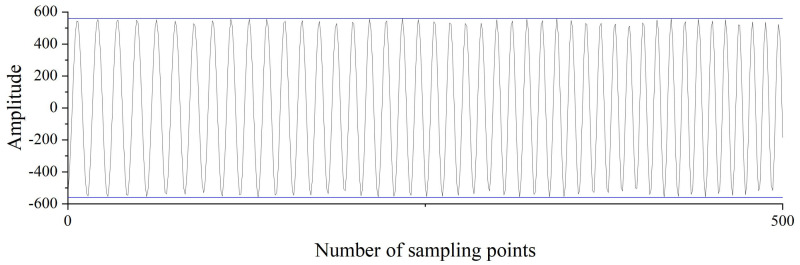
Frequency−varying signal after a dynamic filter.

From Figure 11, the passband gains of the filters in each gear are equal, and there will be no sudden change in amplitude when switching gears. Moreover, with this design, the phase responses of each gear are completely equal, and there is no sudden phase change when switching gears. As shown in Figure 12, a frequency sweep of 4–36 MHz is performed, and the filtered signal does not produce sudden changes in phase and amplitude.

**Figure 13:**
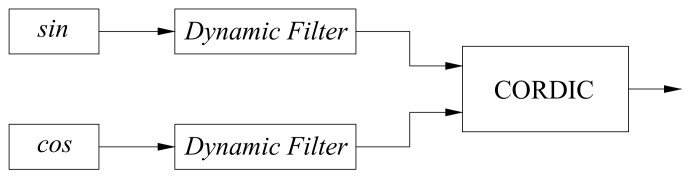
Schematic diagram of a hardware−in−the−loop simulation to prove that the filter does not cause phase abrupt changes.

**Figure 14:**
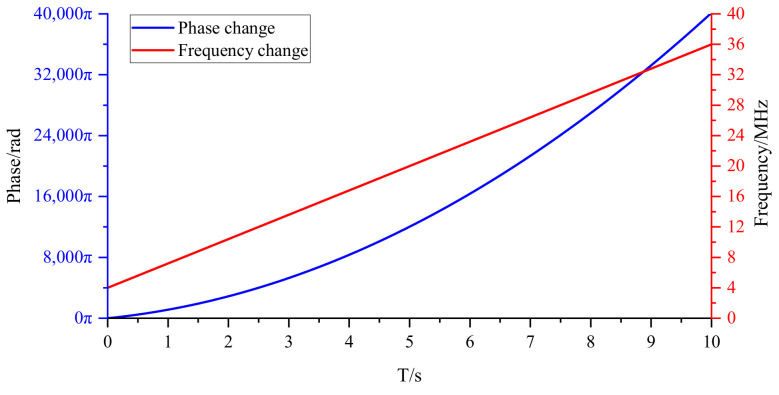
The phase of the quadrature signal whose frequency varies from 4 to 36 MHz after passing through the dynamic filter.

In order to prove that the filter will not cause a sudden change of phase when the gear changes, the following hardware−in−the−loop simulation experiment is designed. Its schematic diagram is shown in Figure 13. A pair of quadrature signals with the same frequency are, respectively, passed through a dynamic filter, and the CORDIC algorithm is used to obtain the phase change of the quadrature signal. As the frequency of the pair of quadrature signals varies from 4 to 36 MHz, their phase changes are shown in Figure 14. The experimental results show that the shift of the dynamic filter will not cause a sudden change of phase.

**Figure 15:**
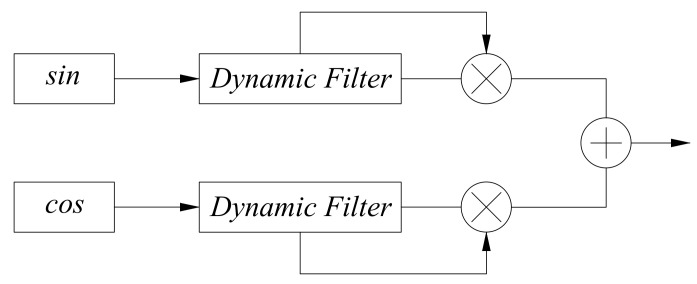
Schematic diagram of a hardware−in−the−loop simulation to prove that the filter does not cause amplitude abrupt changes.

**Figure 16:**
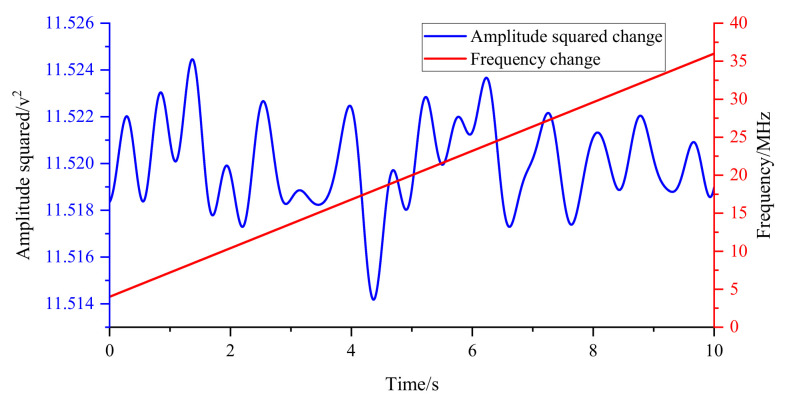
The amplitude of the quadrature signal whose frequency varies from 4 to 36 MHz after passing through the dynamic filter.

Similar to proving that the filter does not cause phase abrupt changes, we design a hardware−in−the−loop simulation experiment as shown in Figure 15. A pair of quadrature signals with the same frequency are passed through a dynamic filter and then squared and summed. The result is proportional to the sum of the squares of the amplitudes of the two signals. The frequency of the pair of quadrature signals is varied from 4 to 36 MHz, and the experimental results are shown in Figure 16. The experimental results show that the gear change of the dynamic filter will not lead to a sudden change in the signal amplitude.

The group delay of an FIR filter with a linear phase response is constant and is one−half the filter order times the sampling time. Because the quality of the reference signal is good and the frequency variation range is not large, ordinary band−pass filtering can be used to improve the signal quality, and the order is usually smaller than that of the dynamic filter. As shown in Equation (17) after the reference signal passes through the band−pass filter and the measurement signal passes through the dynamic filter, the reference signal will lead by Δt. Among them, $n_{1}$ is the order of the dynamic filter, and $n_{2}$ is the order of the ordinary band−pass filter. $t_{s}$ is the sampling time.

(17) $$\Delta t=\frac{\left(n_{1}-n_{2}\right)\times t_{s}}{2}$$

**Figure 17:**
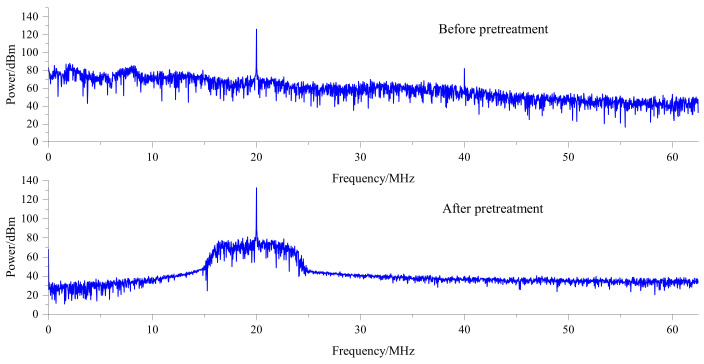
Spectrum of the measurement signal before filter and measurement signal after filter.

The measurement signal before the filter and the measurement signal after the filter are collected in the FPGA. Subsequently, the signal−to−noise ratio of measurement signal before filter and measurement signal after the filter is calculated. The results are shown in Figure 17. Since the ideal measurement signal is a sine wave with a single frequency, the frequency corresponding to the maximum value in the spectrum is taken as the frequency of the useful signal, and the signals of other frequency components are regarded as noise. The ratio of effective signal power to the noise power in the signal is taken as the signal−to−noise ratio. The signal−to−noise ratio of the measured signal is increased from 10.34 dB to 36.20 dB because the dynamic filter attenuates the power of the out−of−band frequency. Experiments show that the preprocessing module can maintain a narrow passband bandwidth and improve the signal−to−noise ratio of the signal when the frequency range of the measurement signal is large.

### 2.2. Phase−Resolving Module Design

The reference and measurement signals are mixed with a pair of fixed−frequency quadrature signals and filtered to remove the 20 MHz dual−frequency laser frequency difference. After the phase difference, the influence of the uncertainty of the dual−frequency laser can be removed and a pair of quadrature signals with equal amplitudes containing only phase information can be generated.

In practical situations, the amplitudes of the reference and measurement signals are different, and there is a laser uncertainty $f^{\prime }$ in the frequency difference. The expressions are shown in Equations (18) and (19).

(18) $$f_{r}=R\sin\left[2\pi \left(f_{1}-f_{2}+f^{\prime }\right)t\right]$$

(19) $$f_{m}=M\sin\left[2\pi \left(f_{1}-f_{2}+f^{\prime }+\Delta f\right)t\right]$$

**Figure 18:**
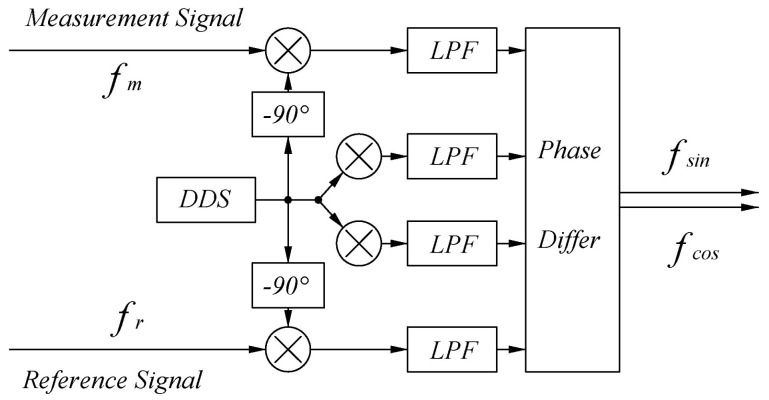
The principal block diagram of the double quadrature lock−in amplification algorithm.

To eliminate the influence of this uncertainty and to solve the problem associated with the amplitudes of the two signals being different, a bi−quadrature lock−in amplification algorithm is introduced to solve the preprocessed reference and measurement signals. Its structural block diagram is shown in Figure 18.

First, the reference and measurement signals are multiplied by the quadrature signal generated by the DDS. Similar to Equations (6) and (7), four mixing signals are formed. Same as Equations (8) and (9), after passing through an LPF with a cutoff frequency of 16 MHz, only the low−frequency part is retained as shown in Equations (20)–(23).

(20) $$f_{r\times sin\;low}=-\frac{1}{2}R\cos\left[2\pi \left(f_{1}-f_{2}+f^{\prime }-f_{d}\right)t\right]$$

(21) $$f_{r\times cos\;low}=\frac{1}{2}R\sin\left[2\pi \left(f_{1}-f_{2}+f^{\prime }-f_{d}\right)t\right]$$

(22) $$f_{m\times sin\;low}=-\frac{1}{2}M\cos\left[2\pi \left(f_{1}-f_{2}+f^{\prime }+\Delta f-f_{d}\right)t\right]$$

(23) $$f_{m\times cos\;low}=\frac{1}{2}M\sin\left[2\pi \left(f_{1}-f_{2}+f^{\prime }+\Delta f-f_{d}\right)t\right]$$

Among them, the frequency $f_{d}$ of the signal generated by the DDS inside the FPGA is equal to the ideal frequency difference $f_{1}-f_{2}$ of the laser. Equation (22) × Equation (20) − Equation (23) × Equation (21) and Equation (22) × Equation (21) − Equation (23) × Equation (20) are calculated, respectively, in the phase difference module.According to Sum−to−product identities, Equations (24) and (25) can be obtained.

(24) $$f_{cos}=\frac{1}{4}RM\cos\left(2\pi \Delta ft\right)$$

(25) $$f_{sin}=\frac{1}{4}RM\sin\left(2\pi \Delta ft\right)$$

The bi−quadrature lock−in amplification algorithm solves the quadrature signals with equal amplitudes and only the Doppler frequency shift term, as shown in Equations (24) and (25) from signals Equations (18) and (19). The problem of laser uncertainty $f^{\prime }$ and the unequal amplitude of the measurement and reference signal is solved.

From the two signals, output by the aforementioned bi−quadrature lock−in amplification algorithm, phase 2πΔft can be solved in the trigonometric function by simply calculating the arctangent of Equations (24) and (25). In this study, the CORDIC algorithm is chosen to realize the arctangent operation in FPGA. The CORDIC algorithm obtains the result by rotating the coordinate axis in a successive approach, which only needs shift operation and addition to calculate the high−precision arc tangent result [21].

## 3. Experiments and Results

**Figure 19:**
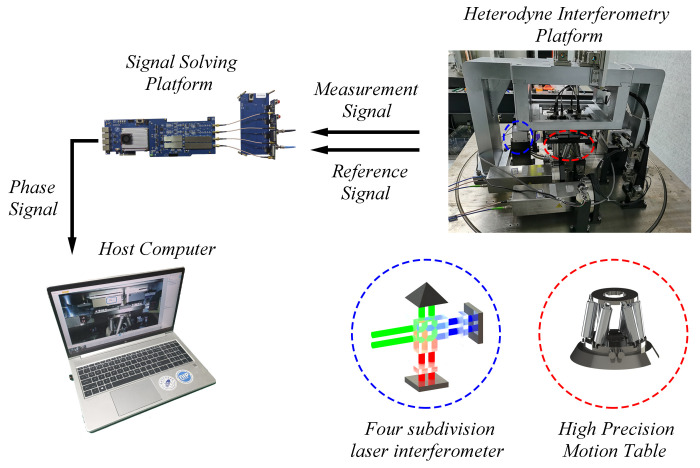
Diagram of the heterodyne interferometry system.

To verify the effect of the algorithm, a heterodyne interferometric measurement system, as shown in Figure 19, is built. Here the motion generated by the high−precision moving stage is measured using a four−subdivided laser interferometer. The measurement and reference signals are calculated from the signal calculation platform into phase signals and then uploaded to the host computer.

**Figure 20:**
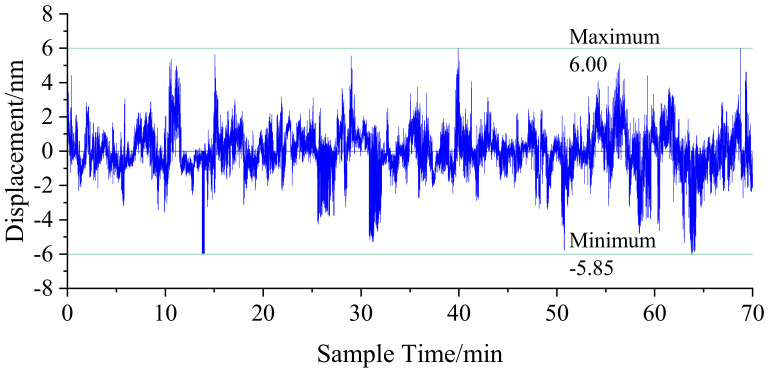
Stability test results.

The long−term phase changes are measured while the motion stage is stationary. The experimental results are shown in Figure 20. Within 70 min, the peak−to−peak value of the change is 11.98 nm. The change is a result of the influence of certain factors, such as environmental interference, table vibration, thermal expansion, and contraction. The results show that the phase−solving algorithm can detect these tiny changes sensitively.

**Figure 21:**
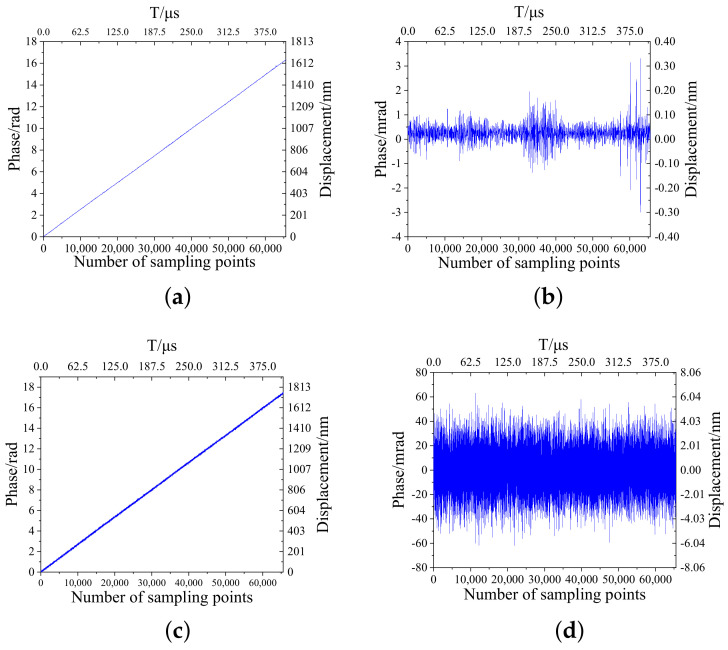
Phase and phase increments with and without dynamic filter for 1 mm/s velocity motion. (**a**) 1 mm/s phase with dynamic filter. (**b**) 1 mm/s phase increment with dynamic filter. (**c**) 1 mm/s phase without dynamic filter. (**d**) 1 mm/s phase increment without dynamic filter.

When the motion table moves at 0.1, 1, 3, 5, 10, and 20 mm/s, a measurement of the phase variation of the heterodyne interference signal processing algorithm with a dynamic filter and general heterodyne interferometric signal processing algorithms are taken. Subsequently, measure the phase change at 65,536 sample points, and calculate the resulting phase difference between each of the two points. Ideally, the phase difference is theoretically equal everywhere; this is due to the extremely short measurement time and very little change in velocity. The phase difference data are analyzed with the results shown in Figure 21 and Table 1.

From the experimental data, we can infer that the error of the solution method without a dynamic filter is approximately 60 mrad and the error is relatively large. The maximum deviation of the phase solution error with a dynamic filter does not exceed 6 mrad, and the maximum peak−to−peak value does not exceed 10 mrad. The maximum deviation does not exceed 1/1024 of the entire cycle, which proves that the first 10 bits of the output result are accurate and achieve the purpose of subdividing 1024 single−cycle phase signals. The equivalent displacement of the phase after 1024 subdivision is 0.15 nm when it is four times the optical path, which proves that the resolution of the system reaches 0.15 nm.

**Figure 22:**
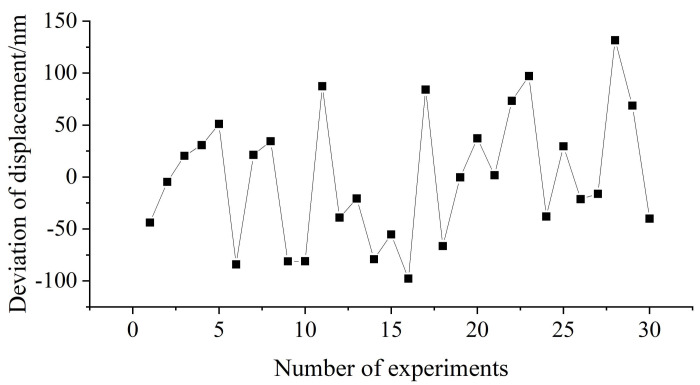
Displacement measurement experiment results.

A displacement experiment was conducted, at a speed of approximately 20 mm/s, the moving table was repeatedly moved by a stroke of 6 μm, and the measurement error was calculated. The results are shown in Figure 22.

The maximum deviation is 131.70 nm, the minimum deviation is 97.80 nm, the standard deviation is 61.76 nm, and the mean value is 3.44 nm. The reason for the large deviation is that the positioning accuracy of the motion stage is low at 150 nm. However, the average deviation is small, which proves that the measured data are accurate.

To verify that the algorithm works at higher frequencies, a signal generator is used to simulate reference and measurement signals to test the algorithm’s performance. The reference signal is a 20 MHz sine wave. The measurement signal was varied at 4–36 MHz. The measurement signal is tested every 1 MHz. The experimental results are shown in Table 2. The Peak−Peak error of the phase increment does not exceed 8 mrad. Experiments show that the algorithm can still guarantee the 1024 subdivision of the single−cycle phase change when the moving stage is fast and the measurement signal frequency is high.

**Figure 23:**
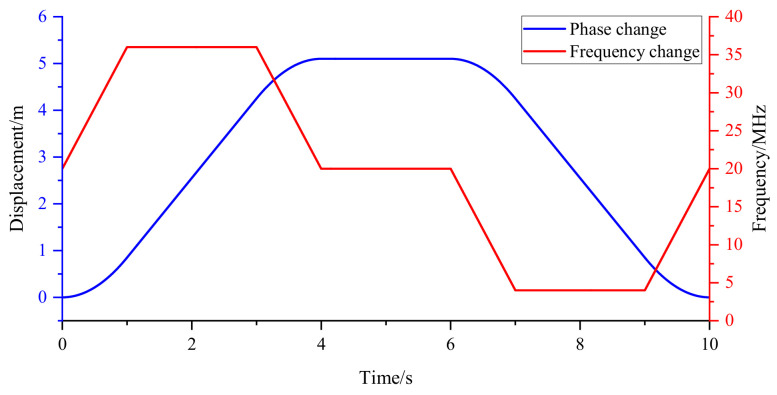
The displacement change calculated by the phase calculation algorithm with dynamic filter when the motion table accelerates and decelerates.

In order to verify the performance of the algorithm when both acceleration and velocity are large, a signal generator is used to simulate the reference and measurement signals when the motion table is in motion. The motion table accelerates to the maximum speed from a standstill and then decelerates to a standstill after moving at the maximum speed for a period of time. The motion table accelerates in reverse to the maximum speed, and after moving at the maximum speed for a period of time, decelerates to a standstill. The frequency change of the measured signal and the calculated phase change are shown in Figure 23. Experiments show that the algorithm can still calculate the phase correctly when the motion table accelerates and decelerates and the frequency of the measurement signal changes greatly.

## 4. Conclusions

On the basis of the characteristics of the heterodyne interferometry system, such as the poor quality of the measurement signal, the uncertainty of the dual−frequency laser, and the different amplitudes between the measurement and reference signals, a subnanometer heterodyne interferometry signal processing algorithm with a dynamic filter is designed. Furthermore, the dynamic filter is designed using variable coefficients. By moving the center frequency of the passband with the main frequency, the bandwidth of the passband can be narrowed under the condition that all valid signals can pass through. The signal−to−noise ratio of the measured signal is increased from 10.34 to 36.20 dB to solve the problems of poor signal quality and low signal−to−noise ratio during the calculation process. Using the bi−quadrature lock−in amplification algorithm, a pair of quadrature signals with the same amplitude including the Doppler frequency shift term are obtained. This solves the problem that the uncertainty of the laser frequency difference and the amplitude of the measurement and reference signals are not equal. When the actual signal generated using the constructed heterodyne interference signal measurement platform is calculated, the final result shows that the maximum deviation of the phase increment does not exceed 6 mrad, reaching 1024 subdivisions for the entire period, and the system resolution reaches 0.15 nm. The algorithm satisfies the requirement of sub−nanometer resolution and can be used as a phase solution algorithm for sub−nanometer heterodyne laser interferometers. When applied to a four−subdivision heterodyne laser interferometer, the measurement speed can reach 10.2 m/s, and the system resolution is 0.15 nm.

## Figures and Tables

**Table 1:** Phase increment errors in phase resolution algorithms with dynamic filters at different speeds.

	Speed (mm/s)	0.1	1	3	5	10	20
Parameter							
Average value (mrad)		0.02	0.24	0.75	1.21	2.54	4.96
Standard error (mrad)		0.22	0.33	0.22	0.39	0.39	0.29
Maximum error (mrad)		1.03	3.24	1.51	5.79	1.30	2.40
Peak−Peak error (mrad)		1.96	6.31	2.66	9.43	2.61	4.79

**Table 2:** Phase increment errors in phase resolution algorithms with dynamic filters at different measuring signal frequency.

**Frequency (MHz)**			**4**	**5**	**6**	**7**	**8**	**9**	**10**	**11**	**12**
Average value (mrad)			−628.2	−589.1	−549.8	−510.6	−471.2	−432.0	−392.7	−353.4	−314.2
Standard error (mrad)			4.63	1.78	1.52	1.64	1.51	1.64	1.89	1.75	1.48
Peak−Peak error (mrad)			7.43	2.48	2.56	2.38	2.14	2.20	5.38	2.37	2.02
Speed (m/s)			−10.20	−9.56	−8.93	−8.29	−7.65	−7.01	−6.38	−5.74	−5.10
**13**	**14**	**15**	**16**	**17**	**18**	**19**	**20**	**21**	**22**	**23**	**24**
−274.9	−235.6	−196.4	−157.1	−117.8	−78.5	−39.3	0.00	39.3	78.6	117.8	157.1
1.55	1.54	1.49	1.58	1.63	1.51	1.49	1.60	1.56	1.41	1.35	1.47
2.20	2.10	2.09	1.94	2.01	2.22	2.14	2.10	2.13	1.95	1.87	1.95
−4.46	−3.83	−3.19	−2.55	−1.91	−1.28	−0.64	0.00	0.64	1.28	1.91	2.55
**25**	**26**	**27**	**28**	**29**	**30**	**31**	**32**	**33**	**34**	**35**	**36**
196.4	235.6	274.9	314.2	353.4	392.8	432.0	471.2	510.5	549.8	589.0	628.3
1.42	1.54	1.71	1.73	1.76	1.65	1.87	1.75	2.02	2.28	3.30	8.26
1.87	2.04	2.27	2.38	2.48	2.35	2.42	2.50	3.17	3.24	4.10	8.00
3.19	3.83	4.46	5.10	5.74	6.38	7.01	7.65	8.29	8.93	9.56	10.20

## Data Availability

Not applicable.
